# Ascorbic Acid—The Little-Known Antioxidant in Woody Plants

**DOI:** 10.3390/antiox8120645

**Published:** 2019-12-14

**Authors:** Karolina Bilska, Natalia Wojciechowska, Shirin Alipour, Ewa Marzena Kalemba

**Affiliations:** 1 Institute of Dendrology, Polish Academy of Sciences, Parkowa 5, 62-035 Kórnik, Poland; mgr.karolina.bilska@gmail.com (K.B.); natalia.wojciechowska@amu.edu.pl (N.W.); salipour@man.poznan.pl (S.A.); 2 Department of General Botany, Institute of Experimental Biology, Faculty of Biology, Adam Mickiewicz University, Uniwersytetu Poznańskiego 6, 61-614 Poznań, Poland; 3 Department of Forestry, Faculty of Agriculture and Natural Resources, Lorestan University, Khorramabad, Iran

**Keywords:** antioxidant system, glutathione-ascorbate cycle, reactive oxygen species, redox status, shrubs, stress, trees

## Abstract

Reactive oxygen species (ROS) are constantly produced by metabolically active plant cells. The concentration of ROS may determine their role, e.g., they may participate in signal transduction or cause oxidative damage to various cellular components. To ensure cellular homeostasis and minimize the negative effects of excess ROS, plant cells have evolved a complex antioxidant system, which includes ascorbic acid (AsA). AsA is a multifunctional metabolite with strong reducing properties that allows the neutralization of ROS and the reduction of molecules oxidized by ROS in cooperation with glutathione in the Foyer-Halliwell-Asada cycle. Antioxidant enzymes involved in AsA oxidation and reduction switches evolved uniquely in plants. Most experiments concerning the role of AsA have been performed on herbaceous plants. In addition to extending our understanding of this role in additional taxa, fundamental knowledge of the complex life cycle stages of woody plants, including their development and response to environmental factors, will enhance their breeding and amend their protection. Thus, the role of AsA in woody plants compared to that in nonwoody plants is the focus of this paper. The role of AsA in woody plants has been studied for nearly 20 years. Studies have demonstrated that AsA is important for the growth and development of woody plants. Substantial changes in AsA levels, as well as reduction and oxidation switches, have been reported in various physiological processes and transitions described mainly in leaves, fruits, buds, and seeds. Evidently, AsA exhibits a dual role in the photoprotection of the photosynthetic apparatus in woody plants, which are the most important scavengers of ozone. AsA is associated with proper seed production and, thus, woody plant reproduction. Similarly, an important function of AsA is described under drought, salinity, temperature, light stress, and biotic stress. This report emphasizes the involvement of AsA in the ecological advantages, such as nutrition recycling due to leaf senescence, of trees and shrubs compared to nonwoody plants.

## 1. Introduction

Plants are immobile organisms and are thus particularly exposed to stress factors of both biotic and abiotic origin. Biotic stressors include all pathogenic organisms, such as viruses, bacteria, and fungi [1], as well as organisms that contribute to a reduction in the photosynthetic area, such as mining insects and herbivorous animals [2]. Currently, the effects of biotic and abiotic stresses are being expanded by global warming, climate change, and environmental pollution [3]. The main abiotic factors that cause stress include UV radiation, high light intensity, low or high temperature, salinity, deficient or excess water, xenobiotics, and heavy metals [4]. All of the above-mentioned stress factors cause a rapid increase in reactive oxygen species (ROS) in the plant, which leads to oxidative stress [5]. The accumulation of high levels of ROS results in oxidative damage to many cellular components and macromolecules, including lipids, proteins, nucleic acids, and sugars [6]. Among ROS, hydrogen peroxide (H_2_O_2_), superoxide anion radical (O_2_^•–^), hydroxyl radical (OH•), and singlet oxygen (^1^O_2_) are produced in plant cells as an inevitable byproduct of processes that occur under physiological conditions linked to oxygen metabolism. In addition to ROS produced in mitochondria, plants must deal with ROS produced in chloroplasts and peroxisomes during photosynthesis and photorespiration. In contrast, discrete changes in ROS concentrations are linked to cellular signaling [7]. ROS promote cellular proliferation, stress acclimation, signal transduction, differentiation and development, metabolic regulation, pathogen defense, physiological cell death, and many physiological responses such as stomata closure, as well as important physiological transitions and switches [8]. For example, ROS are formed in seeds at every stage of their development, i.e., during embryo development, maturation, desiccation, imbibition, the mobilization of storage materials, and germination [9]. Thus, the dual role of ROS in seed physiology depends on their concentration. Notably, to explain this polarity, a model of the oxidative window specifying the ROS concentration range required for the initiation of germination was developed [10].

To ensure cellular homeostasis and minimize the negative effects of ROS overproduction, plant cells have developed a complex antioxidant system. The enzymatic antioxidative system includes enzymes such as superoxide dismutase (SOD; EC 1.15.1.1), catalase (CAT; EC 1.11.1.6), and glutathione reductase (GR; EC 1.6.4.2), as well as plant-specific ROS-scavenging enzymes such as ascorbate peroxidase (APX; EC 1.11.1.11), monodehydroascorbate reductase (MDHAR; EC 1.8.5.1), and dehydroascorbate reductase (DHAR; EC 1.8.5.1). The nonenzymatic antioxidant system mainly includes low-molecular-weight and water-soluble compounds such as ascorbate (Asc) and glutathione [11], as well as fat-soluble α-tocopherol [12]. In addition, numerous polyphenols, such as kaempferol, quercetin, luteolin, myristin, and catechin, have strong antioxidant properties [13].

Ascorbic acid (AsA) and glutathione may be contemplated as the leading antioxidants and constituents of redox signaling; however, another group of biomolecules that should be mentioned in this context are polyamines (PAs), implicated in many of the AsA functions, such as nitrogen recycling and woody plant stress responses [14]. PAs are positively charged molecules protecting the cell against oxidative damage, both directly and indirectly. Directly, they function as antioxidants themselves, thus scavenging free radicals, and indirectly, they have been reported to adjust the levels of enzymatic and/or non-enzymatic antioxidants inside the plant cell. Thus, a rise in PA concentration frequently correlates with an increase in stress resistance, mostly in woody plants like grapevine. However, on the other hand, PA catabolism is one of the main suppliers of H_2_O_2_ to the total H_2_O_2_ cell content and mostly to the cell apoplast [15]. In general, the redox buffering capability of the apoplast is low, regardless of the existence of further antioxidant molecules, such as PAs and flavonoids, and mainly depends on the AsA content due to the lack of glutathione and nicotinamide adenine dinucleotide phosphate [16]. When spermidine-treated grapevine cells/tissues were incubated with AsA and/or PA oxidase potent inhibitors, no evident apoplastic H_2_O_2_ or its visible effects, such as stomatal closure, were detected by light/fluorescence microscopy and by scanning/transmission electron microscopy, thus revealing a significant role for PAs and related antioxidants, such as AsA, in drought and salinity resistance [17]. Polyamine homeostasis plays a dual role by both accumulating and lessening the content of H_2_O_2_ within the cell, thus preserving a delicate line of equilibrium among their molecular contents, conferring plant stress cross-tolerance. Since the maintenance of proper H_2_O_2_ contents within the woody plant cells is of extreme significance for preserving normal developmental procedures and fighting plant stress conditions, an efficient ROS scavenging mechanism by AsA, glutathione, PAs, or other scavenging metabolites, along with the maintenance of suitable metabolite contents, is crucial for plant sustainable growth and survival.

## 2. Synthesis and Structure of AsA

Ascorbic acid, abbreviated as AsA, AA, Asc, and AscH_2_, is vitamin C, a small, 6-carbon molecule. In plants, AsA can be formed through several pathways, including D-glucose, L-galactose, uronic acid, L-gulose, and myo-inositol pathways [18]. The synthesis and accumulation of AsA are particularly sensitive to light, especially the red-to-far-red light ratio, which is directly related to the interaction between photosynthetic and respiratory electron transport chains [11]. L-ascorbic acid (C_5_H_8_O_6_) is the γ-lactone of 2,3-dehydro-L-gulonic acid, known as L-threo-hex-2-enono-1,4-lactone. AsA has strong reducing properties because the moiety between its C2 and C3 carbons easily emits two protons and electrons, becoming a dehydroascorbic acid (DHA) diketone group.

### 2.1. From AsA to DHA—the Oxidation Path

**Figure 1:**
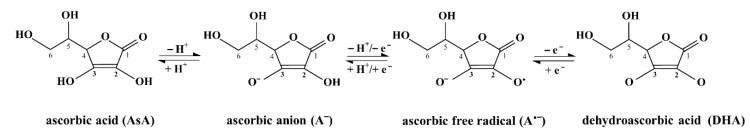
The transformation of ascorbic acid (AsA) into dehydroascorbic acid (DHA). The transfer of protons (H^+^) and electrons (e^–^) is indicated near the arrows. The structural formula of two intermediates, namely, ascorbic anion (AH^–^) and ascorbic acid radical (A^•–^), allow changes during three stages of oxidation. The γ-lactone-ring carbons have been numbered (C1–C6) to indicate the endiol group located between C2 and C3.

A good antioxidant is defined as a nontoxic compound existing in an oxidized form, which can be easily regenerated to the reduced form by the acquisition of electrons from another molecule. Based on the transfer of electrons and protons, cellular metabolic reactions widely use AsA as an electron donor, causing AsA oxidation to DHA. The oxidation of AsA occurs gradually in the three following stages (Figure 1). The first stage of AsA oxidation is the formation of an ascorbic anion (AH^–^). In the second stage, AH^–^ is converted to ascorbic free radical (A^•–^). In the third stage, A^•–^ spontaneously disproportionates, thus generating DHA [19]. DHA can be hydrolyzed to inactive 2,3-diketogulonic acid, which is further degraded to oxalic acid because a high concentration of DHA is toxic [20].

### 2.2. The Continuum of AsA-DHA-AsA Switches

**Figure 2:**
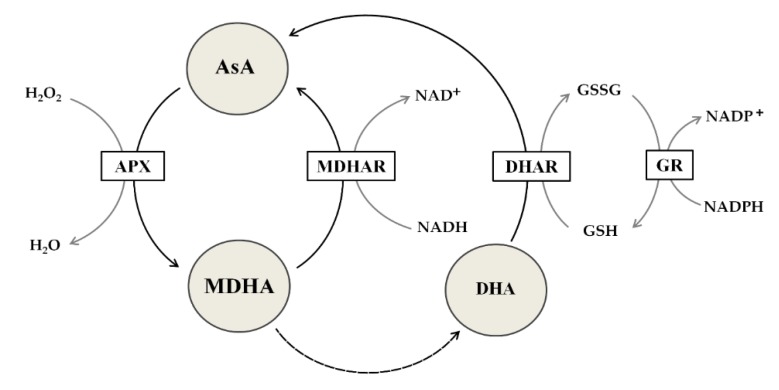
The ascorbate-glutathione cycle. Detoxification of hydrogen peroxide (H_2_O_2_) into water (H_2_O) occurs due to the action of ascorbate peroxidase (APX). APX uses ascorbic acid (AsA) as an electron donor to produce monodehydroascorbate (MDHA), which is then reduced to AsA by monodehydroascorbate reductase (MDHAR), whose cofactor is nicotinamide adenine dinucleotide (NADH). Dehydroascorbate (DHA), which is formed by disproportionation, is reduced to AsA by the action of dehydroascorbate reductase (DHAR), the cofactor of which is reduced glutathione (GSH). As a result of DHAR’s activity, GSH is oxidized to glutathione disulfide (GSSG), which is then reduced to GSH as a result of the activity of glutathione reductase (GR), which acquires electrons from nicotinamide adenine dinucleotide phosphate (NADPH).

The redox potential of AsA enables interactions with ^1^O_2_, O_2_^-•^,OH•, glutathione radicals, and tocopherol radicals [21]. AsA can directly neutralize ROS and can participate in the repair of oxidized organic molecules. Many primary and secondary metabolites can act similarly as AsA and glutathione, but unlike these compounds, AsA and glutathione interact with many components, including specific enzymes and signaling pathways [11]. Their oxidized forms are relatively stable, and their reduction is highly efficient due to enzymatic systems based on electron transporters such as nicotinamide adenine dinucleotide phosphate (NADP). The important pathway that involves both molecules is the Foyer-Halliwell-Asada pathway, also known as the ascorbate-glutathione cycle [21]. The contribution of AsA to the detoxification of H_2_O_2_ is based on APX activity [19], which forms MDHA (Figure 2). DHAR can regenerate DHA to AsA using reduced glutathione (GSH). Thus, cooperation between AsA and GSH is essential in cyclic redox reactions (Figure 2). Interestingly, AsA can produce intermediates with lower toxicity than glutathione radicals [22]. Thus, AsA is the best candidate reductor, which merits further study, including detailed research comparing the role of AsA in woody plants to that in nonwoody plants needed to be explained in detail.

## 3. Functions of Ascorbate

Asc is a cofactor for violaxanthin de-epoxidase, which is involved in photoprotection through the xanthophyll cycle, both in woody [23] and nonwoody plants [24]. As shown in a meta-analysis related to the photoprotection of plant functional groups and plant growth types, the Asc concentration of total chlorophyll (expressed in mmol of Asc per mol of chlorophyll) in nonwoody plants is not detectable, while that in woody plants can be up to 25,000 in shrubs and half that value in trees [25]. Foliar photoprotective defense systems in trees are relevant in the context of climate-originating water deficits and changes in temperature to sustain photosynthetic activity [26,27,28]. These photoprotective mechanisms are extremely important in the sun-exposed leaves of woody plants differing in photooxidative stress tolerance [29,30,31]. High levels of AsA and photoprotective pigments were reported in sun-exposed beech leaves [32], apple fruits [33], and high-light-demanding species [34], emphasizing the dual protective role of AsA in ROS scavenging and the activation of xanthophyll-cycle enzymes in leaf photoprotection. Hansen et al. [35] suggested that the accumulation of leaf pigments and antioxidant systems together might also be a prerequisite for shade tolerance with ecological implications in a mixed beech/oak forest. The three connected categories, that is, the xanthophyll cycle, photoprotection, and photooxidative stress, are overrepresented in woody plant studies.

AsA is also necessary for the anthocyanin biosynthetic pathway, the activity of enzymes involved in the biosynthesis of flavonoids and glucosinolates in plants [36], and for enzymes involved in the biosynthesis of plant hormones such as abscisic acid (ABA), gibberellins (GA), and ethylene [37,38], indicating that AsA is a multifunctional metabolite. Antioxidants are no longer considered only electron donors, as they can modulate cellular signals and gene expression. By impacting the synthesis and concentration of hormones, AsA modulates hormone-dependent signaling pathways [39,40] and together with glutathione, affects redox signaling [11]. Asc alone can impact gene expression by modulating the intracellular cAMP pool [41]. APX activity-dependent AsA to DHA switches have been reported to modulate secondary cell wall-related gene expression in cotton [42]. Importantly, Asc is involved in the regulation of transcripts encoding the proteins of photosynthetic and respiratory electron transport chains [43]. Further studies are needed because the modulation of antioxidant responsive elements by AsA has been studied exclusively in humans.

### 3.1. The Involvement of Asc in the Development of Plants

Studies concerning Arabidopsis vitamin C (vtc)-deficient mutants producing 10–30% of wild-type Asc levels revealed that Asc regulates plant growth [44]. AsA is involved in the cell’s transition from phase S to G1 [20] and the cross-linking of cell wall components [45], and is therefore important in growing tissues. It was shown that double cytosolic APX-silenced rice plants produced a semidwarf phenotype [46], whereas thylakoid APX wheat mutants exhibited decreased photosynthetic activity [47]. Both studies revealed that compensatory antioxidant mechanisms were activated in APX mutants, but deficiency in AsA to DHA switches contributed to growth abnormalities. Asc accumulates primarily in photosynthetically active tissues, meristematic tissues, flowers, young fruits, root tips, stolons, and tubers. The level of Asc depends on the developmental stage, diurnal rhythm, light, and many external factors that introduce stress [44]. Diurnal variation in the transcript levels of genes involved in AsA synthesis was reported in tomato [48] and Arabidopsis [49]. Apart from the definite involvement of Asc in the regulation of whole-plant development [50], Asc modulates the growth of woody plants in further manners.

#### 3.1.1. Development of Woody Plants

The level of Asc depends on plant age. For example, the younger the mango tree, the more AsA will be in its fruits [51]. The level of Asc in leaves and fruits also differs along the vertical canopy profile. High Asc levels were detected in European beech leaves of the top and middle canopy layers, whereas the Asc level was halved in leaves of the bottom layer [52]. Fruit peels from the exterior of the canopy of three apple cultivars contained higher AsA levels than those from the interior of the canopy [53]. In nonwoody plants, the Asc distribution across canopy profiles is less studied and probably less significant. For example, DHA levels were found to differ between the second, fourth, and sixth main stem leaves of soybean, whereas no such difference was detected in AsA levels [54]. In trees, the level of Asc also depends on the needle age. Adult needles of *Pinus pinaster* seedlings contained several times more Asc than juvenile needles [55]. The level of the AsA-based antioxidant response is also linked to the age of trees. Turfan et al. [56] demonstrated that Anatolian black pine needles from over-500-year-old trees contained the highest concentrations of APX among all age classes of younger trees. The selection of commercial cultivars and even experimental hybrids that are extremely rich in AsA and other valuable biochemicals is a trend in crop improvement in terms of the bioavailability of human wellness compounds, such as vitamin C [57,58]. Natural breeding resulting in changes in the expression of bioactive molecules, such as pyrophosphate, can improve the AsA content in food products [59]. Similarly, light manipulation can elevate AsA levels. Light enhances the expression of L-galactono-1,4-lactone (GalLDH), an enzyme catalyzing the final step in the AsA biosynthesis pathway [60], which was found to be related to extremely high AsA levels in chestnut rose [61]. However genetic engineering focused on enzymes of AsA biosynthesis pathway seems to be more promising. Bulley et al. [62] suggested that the overexpression of GDP-L-galactose guanyltransferase, another enzyme involved in AsA synthesis, can contribute to significant increases in fruit AsA. Transcription factors involved in modulating the expression of genes linked to the AsA biosynthesis pathway should also be of future interest [63]. For example, a WRKY transcription factor involved in drought tolerance promotes AsA accumulation in pear [64]. Combined transcriptomic and metabolomic approaches demonstrated that Asc regulation is complex and linked with whole-plant metabolism and pathways related to stress responses [50]. In this context, vitamin C-enriched crops are a prospect for the distant future.

#### 3.1.2. Senescence

Asc delays leaf senescence [65], which is an oxidative process. Thus, decreased Asc levels were reported in aged tobacco [66] and spinach leaves [67]. Barth et al. [68] demonstrated that the Arabidopsis vtc1 mutant enters senescence faster than the Arabidopsis wild-type. Recently, ROS-mediated leaf senescence was confirmed in APX-deficient rice mutants [69]. Many woody plant studies have shown that changes in APX activity are linked to apricot [70], ginkgo, birch [71], and beech [72] leaf senescence. Leaf senescence limits yield in certain crops, whereas in forests of deciduous trees, leaf senescence enables nutrition recycling in ecosystems.

#### 3.1.3. Aging

The postharvest quality loss of nonwoody plants is followed by decreasing AsA [67]. Interestingly, in the fruit peel of tangerine x sweet orange, the level of APX decreased during the postharvest aging process and increased during drought [73]. The early stages of *Ginkgo biloba* L. leaf expansion were accompanied by increased activity of APX [74], emphasizing the involvement of Asc in the maximization of leaf-space to increase both the interception of light and photosynthesis. Studies on photosynthetic activity in nonwoody plants frequently focus on stress conditions such as water deficits [75] or mineral element imbalances [76]. Studies on Arabidopsis vtc mutants revealed that AsA contributes to photosynthesis activity [77], but is not essential for photoprotection [78]. In contrast, Asc-related photoprotection in woody plants is well documented [27,28,29,32].

#### 3.1.4. Root development

Asc is crucial for root growth and development. Asc stimulates the elongation of roots [79] and is involved in quiescent center (QC) organization and activity in both nonwoody and woody plants [80,81,82]. The maintenance of the root QC is accompanied by a low Asc content and high ascorbate oxidase (AO) activity, resulting in the dominance of DHA in the Asc pool [80]. Interestingly, studies of poplar revealed that AsA and GSH are transported from mature leaves to the root tips in a dissimilar manner. GSH is withdrawn from the phloem along the entire transport path, whereas the entire AsA pool is delivered to the root tips [83]. Interestingly, the phloem transport of AsA might be considered a shoot-to-root signal coordinating whole woody plant growth and development [83], because, in nonwoody plants, the downstream phloem transport of AsA depends strictly on the AsA content in leaves [84].

#### 3.1.5. Fruit Development

Fruit development and maturation is considered to be an oxidative phenomenon, and the extent of oxidative stress depends on the action of antioxidants, i.e., AsA. Santos et al. [85] determined that APX was involved in increasing ROS removal in coffee fruits from trees grown at high altitudes. The MDHAR transcript was reported in overripe acerola (*Malpighia glabra*) fruits, whereas the expression of DHAR was the highest in the intermediate stage of acerola fruit ripening [86]. Fruits in particular contain large amounts of Asc, which is the best source of vitamin C for animals. Tree transcriptome sequencing revealed that the expression of genes involved in the L-galactose pathway is linked to high Asc levels in acerola fruit [87]. Large-sized mandarin fruits contain high AsA levels [88], whereas fruit color and fruit size had no detected effect on the Asc content in sweet cherry fruits [89]. Among fruits, berries are extremely rich in Asc. Sea buckthorn berries are known as the “king of vitamin C” because the AsA synthesis pathway is enriched in this species, with the uronic acid pathway characteristic to animals [90]. Aronia berries contain 31.85 mg of Asc per 100 g of fresh fruits [91], whereas camu-camu berries contain up to 38 g of AsA per 100 g of dry pulp [92]. Brazilian native fruits contain 9.351 mg AsA per 100 g of butia, with approximately 0.1 mg AsA in Araça and pitanga [93]. The level of Asc (mg/100 g of fresh fruit) ranges from 85.9 to 104.5 in guava, 17.5 to 23.6 in mango, and 79.2 to 82.2 in papaya [94]. The levels of synthesized and recycled AsA in leaves and fruits are modified by light. For example, light affected the AsA levels in the apple peel and leaf but not in the apple flesh [95]. Pear fruits grown in the dark contained lower Asc levels than fruits grown in the light [96]. Similarly, AsA accumulated in the peel of shaded fruit [97]. Interestingly, tomato fruit shading directly reduced the Asc, AsA, and DHA contents, whereas tomato leaf shading increased the DHA content but had no effect on AsA [98]. Molina-Delgado et al. [99] demonstrated that on-tree apple ripening is linked to endogenous levels of Asc and CAT, which emphasize apple maturity patterns exhibited in orchards. In fruits located near the photooxidatively stressed leaves of orange trees, the activity of antioxidants, including APX and MDHAR, was significantly elevated [100], indicating more efficient ROS removal. Low night temperatures, water stress, and the application of nitrogen fertilizer during growth decrease Asc levels, whereas a high light intensity and fruit acidity increase Asc levels in fruits [101]. Asc accumulation is also induced by light in fruits of nonwoody plants [102]. The higher the expression of genes involved in AsA biosynthesis, the higher the AsA content in developing pepper [103], tomato fruits [104], and grape berries [105], and during fruit ripening [105,106]. Oxidative stress is alleviated by the action of antioxidant enzymes such as APX. A 3–4-fold increase of APX transcripts was found during pepper fruit ripening [107]. However, the fruit aging process results in a decrease in APX activity [108]. Additionally, Asc is involved in fruit softening [109].

#### 3.1.6. Bud Development

Asc participates in the regulation of bud development. APX activity was found to be related to the transition from endodormancy to ecodormancy in Japanese pear buds [110]. As a consequence of H_2_O_2_ production during flower bud development, APX activity was considered one of the markers of flower bud development stages in lemon [111] and Japanese pear [110]. Asc protects sweet cherry buds during para- and endodormancy phases [112]. APX is considered one of the markers for sex determination [113]. APX is a female-specific protein in tung tree flowers [114] and is male-specific in Pistacia [115]. AsA promotes pea bud growth by interacting with hormones and light [116]. High AsA levels are important for soybean floral bud initiation [117,118] and anther development in cotton [119].

#### 3.1.7. Seed Development

Asc is essential in seed embryogenesis and the seed filling phase [19,120]. The AsA concentration was much higher in embryonic axes of developing *Acer platanoides* L. and *Acer pseudoplatanus* L. seeds [120] and mature *Acer saccharinum* L. embryonic axes than in cotyledons [121]. A gradual decrease in the Asc pool was reported in cotyledons of developing *A. platanoides* [120] and *Fagus sylvatica* L. seeds [122]. At early stages of seed desiccation, an elevated Asc pool was detected in the embryonic axes of *A. saccharinum* [121]. However, desiccated seed tissue contains low amounts of Asc because of the limited water content and metabolism shutdown [11]. The regeneration of AsA from DHA is GSH-dependent and is realized by the action of DHAR, which was reported as important to *A. saccharinum* seed viability [123]. The accumulation of Asc in fruits affects seed viability, the germination capacity, and seedling establishment, thus strongly impacting woody plant reproduction [19]. Asc also affects seed development in nonwoody plants at many stages. For example, Asc was found to be important for wheat kernel maturation [124] and ABA-dependent barley seed germination [125]. APX activity was shown to be important in soybean during the seed filling phase [126] and in rice seed germination and seedling establishment [127]. AsA was shown to be a major ROS detoxifying antioxidant during the accelerated aging of sunflower seeds [128] and cotton seeds [129], during lupine seedling growth [130], during pine hypocotyl aging, and in the pine hypocotyl axis [131]. The above data suggest that Asc acts as an important antioxidant in seed production in all plant organisms. Seed development and maturation can last up to 5–6 months in woody plants; therefore, antioxidant protection is essential. Thus, by regulating the redox status in seeds, AsA contributes to the reproduction of woody plants.

### 3.2. The Involvement of Asc in Response to Abiotic Stress

The efficient neutralization of H_2_O_2_ relies on fast AsA oxidation and the action of APX. APX isoforms are localized in the cytoplasm, peroxisomes, and chloroplasts. The increased stability of chloroplastic APX compared to cytosolic APX (cAPX) results from a unique loop in the enzyme structure of chloroplastic APX [132]. However, analyses of Arabidopsis mutants lacking cAPX demonstrated that the chloroplastic H_2_O_2_-scavenging system completely failed without cAPX [133], showing the interrelationship of APX isoenzymes in diminishing oxidative stress. The activity of APX was demonstrated to be important in plant responses to salt, heat, cold, light, drought, and oxidative stress [134]. Asc is an important antioxidant in the acclimation process to high light [135,136]. Arabidopsis studies revealed that an increased Asc content can limit the deleterious effects of high light- and high temperature-induced oxidative stress [137]. AsA alleviates heat stress-induced damage in tall fescue [138] and strawberry [139]. The inducible expression and activity of APX isoforms differed in sand pear cultivars exposed to heat stress, reflecting their tolerant and sensitive attributes [140]. Jin et al. [141] reported that a heat-induced increase in the APX activity in the leaves of *Euonymus japonicus* seedlings coincided with cyclic electron transport around photosystem I. AsA to DHA transformation protects against stress conditions in both woody and nonwoody plants.

#### 3.2.1. Ozone

Ozone (O_3_) is a toxic gas and powerful oxidation agent encountered by plants. The 30–50 ppb O_3_ concentration significantly reduces plant biomass production and reproduction [142]. Crowther et al. [143] estimated that there are more than 3.04 trillion trees on Earth. In this context, trees might be considered important scavengers of harmful atmospheric trace gases, mainly O_3_, in a process that robustly involves Asc. For example, the level of apoplastic Asc in Scots Pine needles was strongly correlated with the level of atmospheric O_3_ during the seasonal course [34]. Additionally, the premonsoon and postmonsoon seasons, as well as air pollution, were reported to affect the level of AsA in several tree species and led to the identification of air pollution-tolerant tree species [144]. The AsA content increased in the leaves of many herbaceous plants, including crops, growing at polluted sites [145,146,147]. However, deciduous woody plants and conifers were more tolerant to O_3_ than herbaceous crops [148]. Although AsA levels shape the baseline of plant stress responses, knowledge concerning the involvement of AA in plant recovery after O_3_ stress has yet to be elucidated [149]. Elevated Asc levels and enhanced AsA oxidation were also reported in O_3_-exposed date palm seedlings [150]. Along with Asc, glutathione plays an essential role in the growth and antioxidant defense of poplar saplings under O_3_ conditions [151]. APX activity and AsA regeneration by MDHAR were indicated as the main determinants of O_3_ tolerance in Euramerican poplar, as they efficiently detoxified ROS in leaves and enabled photosynthesis [152]. Hypersensitivity to O_3_ was detected in the Arabidopsis mutant vtc1, which contains low Asc concentrations [68]. Nonwoody plants exposed to O_3_ exhibit leaf damage, decreased rates of CO_2_ assimilation, and a decline in photosynthesis [153], which affect crop biomass. Thus, the selection of crop cultivars [154] and varieties [155] resistant to elevated O_3_ levels is needed. However, the involvement of nonwoody plants in reducing air pollution and O_3_ scavenging is low because the world’s forests absorb a third of global pollutant gas emissions every year. Furthermore, Matyssek et al. [156] reported clear interactions between O_3_ and drought in forest trees. O_3_ levels affecting stomatal function decrease drought tolerance. In contrast, a drought period followed by O_3_ stress may harden forest trees the impacts of O_3_, resulting in important ecological adaptation. Such a phenomenon is not possible in annual plants.

#### 3.2.2. Drought

Water shortage causes the overproduction of ROS and oxidative stress in plants. Studies concerning monsoon seasons revealed that recovery from water stress mimicking the dry season in rice depends on antioxidants, mainly the ascorbate–glutathione cycle [157]. Drought stress applied to mature Aleppo pine trees induced the downregulation of transcripts linked to photosynthesis and ROS removal via the ascorbate-glutathione cycle and the upregulation of ROS removal via AsA-independent thiol-mediated pathways [158]. Drought stress inhibited the biosynthesis and decreased the concentration of AsA in soybean plants [159]. In response to the application of identical stress conditions, including drought, air warming, and soil with different pH levels, the leaves of three oak species had different antioxidant response levels. The highest Asc and AsA levels were reported in the leaves of *Quercus robur* seedlings, while *Quercus pubescens* was characterized by lower levels of total foliar Asc [160]. Interspecies differences in the levels of Asc forms were also detected in *Acer* seeds [120]. In regions characterized by dry periods, trees suffer from climate-originated oxidative stress, and the range of antioxidant responses of the ascorbate-glutathione cycle is species-specific [161]. Provenance-specific reactions to drought stress based on the synthesis of osmolytes and increasing levels of ROS scavengers, including AsA, were observed in Douglas fir [162]. The activities of APX, MDHAR, and DHAR increased as drought stress progressed in the leaves of wild almond, whereas rewatering downregulated their activity [33]. The cAPX transgene from pea enhanced water stress tolerance in transgenic plums [163]. This is a promising result because water shortages are a worldwide problem under global climate change. In general, APX activity increases in drought-tolerant tissues and species. In European larch seedlings, the APX activity was doubled under water stress and nearly 4 times higher under salinity stress. Nonwoody plant studies revealed that APX protects organelles against wounding-originated oxidative damage [164], limits salt stress-induced damage [165], and enhances resistance to chilling stress [166]. Arabidopsis vtc1 mutants exhibit increased sensitivity to salt stress [167]. Under salinity stress, multiple antioxidants, including APX, are elevated [168]. For example, DHAR overexpression enhances salt tolerance in tobacco [169]. Salinity reduces the AsA level in the roots and leaves of pistachio [170], emphasizing that AsA is involved in the response to external osmotic pressure around the roots in the soil, as well as the response to the toxic effect of salt ions accumulating mainly in leaves. AsA synthesis in roots regulates the response to salt stress by elevating ROS scavenging [171]. Many areas in the world are affected by excess salinity, but in contrast to crops, little progress has been made in improving the salt tolerance of forest tree species.

#### 3.2.3. Pollution

Adaptations to metal-originated oxidative stress, including increased ascorbate-glutathione cycle activity immediately after the introduction of stress conditions, is essential for potential plant-origin bioaccumulators. In a heavy-metal-polluted environment, the use of a specific effluent can lead to the identification of different tree species that are adequate for phytoremediation [172]. The Asc level was suggested as an indicator of the environmental pollution degree after elevated Asc levels were observed in pine needles collected from stands affected by industrial pollution [173]. The air pollution tolerance index (APTI) calculated using the AsA content has been used to select plants that can be used for biomonitoring air pollution [146]. As a reaction to oxidative stress, increased APX activity was found in the roots of acacia seedlings growing in oil-contaminated soils [174] and the leaves of osier grown on Cr-rich tannery waste [175]. Moreover, studies concerning European beech leaves and Norway spruce needles revealed that the mechanisms of phytogenic reduction and the emission of mercury in grass plants and trees are similar and depend on the AsA concentration [176,177]; however, in the context of their leaf biomass, trees are clearly more efficient emitters than grass plants [177].

#### 3.2.4. Cold

Studies of eight clones of *Hevea brasiliensis* Muell. Arg. revealed that photosystem II is extremely sensitive to cold and that ROS elimination is a crucial step for determining chilling tolerance. Strong APX, DHAR, and MDHAR activities during recovery are characteristic of cold-tolerant clones [178]. For example, the levels of Asc and other antioxidants were higher in cold-tolerant rubber tree clones than in cold-sensitive clones in response to applied cold stress [179]. Cold and salt stress upregulated the transcription and increased the activity of MDHAR and DHAR in acerola leaves [86]. Seasonal changes in the Asc level, which was highest in the winter and lowest in the summer, were detected in each needle class of *Pinus sylvestris* L. [180]. Asc was up to 50-fold more abundant than other antioxidants, including glutathione, tocopherol, and carotenoids, playing a key role in *Quercus ilex* L. protection against photooxidative stress during winter [181]. Similar to Asc, the xanthophyll cycle also exhibits a photoprotective role during the cold acclimation of needles in winter [182], thus advantaging evergreen shrubs and trees.

#### 3.2.5. Acid Rain

Another environmental factor harmful to woody plants is acid rain, which exposes trees to toxic substances that are gradually released from the soil. Acid rain impacts seed germination, seedling growth, and photosynthesis in forest tree species [183]. Interestingly, seedlings of acid rain-sensitive and tolerant tree species exhibit modified APX expression [184], indicating that AsA action differentiates these two phenotypes. Because of the negative effect of acid rain on crop performance, simulated acid rain studies also concern nonwoody plants. For example, the lower the pH, the higher the decrease in the AsA content observed in peppermint leaves [185]. Finally, a study investigating the combined effects of AsA application and simulated acid rain on Persian maple revealed that elevated APX activity is crucial in plant defenses against acid rain stress [186]. Considering that AsA oxidation is essential to alleviate acid rain effects and that the AsA pool is higher in trees and shrubs than in nonwoody plants, woody plants seem to be better protected against acid rain.

### 3.3. The Involvement of Asc in the Response to Biotic Stress

Asc acts in bacterial, viral, and fungal infections affecting woody plants. The Asc level differed in the roots and leaves of Eucalyptus seedlings at several stages of infection with soil-borne water mold *Phytophthora cinnamomi* [187]. Studies concerning the fungal disease of citrus trees caused by *Phoma tracheiphila* (Petri) Kantschaveli and Gikashvili revealed that APX activity was up- and downregulated depending on the type of rootstock in resistant and susceptible species, respectively [188]. Viral infection of apricot seeds resulted in decreased activity of enzymes of the ascorbate-glutathione cycle and decreased germination capacity [174]. APX was involved in different antioxidant responses to bacterial infection in young and old leaves of *Pyrus communis* cv. Conference [189]. AsA participated in the dynamic antioxidant response of Norway spruce phloem attacked by *Ips typographus* L. [190]. Thus, decreasing the availability of AsA by AO activity might be a defense strategy in poplar against leaf-chewing insects [191]. In contrast, the AsA content increased in cabbage leaves after flea beetle attack [192], indicating differences between the insect attack responses in woody and nonwoody plants. Arabidopsis vtc1 mutants were more susceptible than the Arabidopsis wild-type to viral infection [193]. Interestingly, vtc1 and vtc2 mutants exhibited enhanced resistance against bacterial infections by *Alternaria brassicicola* [194]. However, fungal infection resulted in declining Asc and the extensive oxidation of the remaining AsA, particularly in these mutants [195], indicating the complexity of the defense response.

#### Mycorrhiza

Studies of *Robinia pseudoacacia* revealed that ROS removal by antioxidants such as APX is additionally enhanced by the presence of arbuscular mycorrhizal fungi symbiosis [74]. Increased APX activity was also observed in tobacco seedlings inoculated with arbuscular mycorrhizal fungi [196] and tomato seedlings under salt stress [197]. APX activity is directly linked to the enhanced growth of shrub seedlings inoculated with mycorrhiza [198] and their drought tolerance [199]. Arbuscular mycorrhiza, which increases plant defenses and disease resistance, is applied as biostimulant to some agricultural and horticultural crops produced at a large scale, and is predominantly used in forest tree nurseries [200]. However, the advantages of arbuscular mycorrhiza are greatest for woody plants in natural environments and result in significant ecological services.

### 3.4. The Effects of AsA Application

Exogenously applied reducing agents strongly affect redox status and viability in oxidative environments [123]. The treatment of pruned stems of incense tree with AsA greatly reduced the H_2_O_2_ postwounding signal, and its effects manifested in a reduced number of vessel occlusions and a lower amount of synthesized sesquiterpenes [201]. AsA displays metal-ligand binding properties. AsA renders titanium (IV) stable in aqueous solution, resulting in the bioactive form of Ti (IV) used to increase crop yields [202]. Under limited micronutrient uptake, foliar Ti-ascorbate sprays improved the vigor, nutritional status, and level of Ti in the leaves of maiden apple trees [203]. AsA foliar applications contributed to increases in the primary root length and height of almond seedlings [204]. Depending on the AsA concentration used, foliar applications of AsA resulted in increases in the leaf area, shoot number, total chlorophyll content, and root dry weight of olive transplants [205]. It was suggested that foliar applications of AsA could be used as a management practice to alleviate the water stress in young peach trees and improve their performance after rewatering [206]. In nonwoody plants, AsA foliar spraying efficiently mitigated the detrimental effects of water stress in maize [207] and wheat [208], and Pb toxicity-induced oxidative damage in wheat plants [209] and faba bean [210]. Asc foliar spraying increased tolerance to water deficit by reducing ROS levels and ROS-induced damage in pot marigold [211], increasing the resistance to salt stress and lipid peroxidation in tomato [212], and affecting millet growth and salinity resistance [213]. Foliar application of AsA could be useful in crop production after the transfer of increased grain yield from small-scale laboratory experiments to large-scale production, because the combination of AsA spraying and nitrogen fertilization is beneficial for grain yield and plant height [214].

In summary, the role of AsA in woody plants to that in nonwoody plants is compared and contrasted in Table 1.

## 4. Conclusions

The antioxidant capacity of AsA is based on the detoxification of H_2_O_2_ and is performed by the APX enzyme and MDHAR and DHAR activities, which regenerate DHA to AsA. AsA is involved in the alleviation of oxidative stress manifested by a rapid increase in ROS following specific developmental events and multiple stress-introducing environmental factors. Recent studies have demonstrated that AsA is involved in root, shoot, leaf, and fruit development in woody plants. In particular, AsA participates in photosystem protection and acts in leaves, enabling the continuation of photosynthesis. Equally, AsA contributes to seed viability and the reproduction of trees. Some studies suggest that Asc can be used for the assessment of photooxidative stress tolerance in trees. Admittedly, AsA is crucial in redox homeostasis, and it has been suggested that its content in trees indicates the degree of environmental pollution, and it is an important player in O_3_ scavenging by trees, which seems to be globally important because O_3_ reduces the plant biomass throughout the whole ecosystem. Many studies have confirmed that, in trees subjected to drought, salinity, heat, cold, and high light intensity, as well as in trees infected by bacteria, viruses, fungi, or insects, the changed redox state can be efficiently restored to homeostasis by the action of AsA and enzymes, enabling DHA reduction. All of the above benefits in tree growth and protection have caused foliar applications of AsA to be used as a management practice in forest nurseries.

## Figures and Tables

**Table 1:** Comparison of the involvement of different forms of ascorbate and enzymes linked to ascorbate metabolism in the regulation of plant growth and development, as well as responses to abiotic and biotic stress, between woody and nonwoody plants.

	Woody Plants	Nonwoody Plants
Plant Development		
synthesis of hormones and flavonoids	[26]	[25,38]
cellular signals	[41]	[40,42]
whole plant growth and development	[83]	[19,50]
vertical canopy profile	[52,54]	[62]
leaf expansion	[74]	
leaf senescence	[69,70,71]	[72]
photosynthetic activity	[30]	[75,76]
photoprotection	[23,29,32]	[27]
xanthophyll cycle	[23,28,29]	[24]
root growth	[83]	[79]
root quiescent center organization	[82]	[80,81]
organ and plant age	[63,64]	[66]
fruit development	[51,87,95,96,97]	[50,102,103]
fruit ripening	[86,99,105]	[53,60,106]
fruit size	[88,89]	[55,56,107]
Asc accumulation in fruits	[90,91,92,93,94]	[58,59,104]
fruit softening and postharvest fruit aging	[51,73,87]	[67,108,109]
cross-linking of cell wall	[45,131]	[79,130]
bud development and dormancy	[110,112]	[116,117,125]
flower development and sex determination	[111,114,115]	[113,118,119]
seed embryogenesis, seed filling phase	[120,122]	[19,124,126]
seed desiccation	[120,121,122]	[19]
seed viability	[123]	[128,129]
germination and seedling establishment	[121]	[127,130]
diurnal rhythm	[44]	[48,49,84]
seasonal changes	[32,180]	
monsoon seasons	[144]	[157]
Abiotic Stress		
ozone	[34,142,150,151,152,156]	[153,154]
oxidative stress	[120,123,142,159,160,161,162]	[46,137,164,210,211]
photooxidative stress	[31,34,100,181]	[47,136,164]
drought stress	[33,61,163,168]	[159,212]
salinity stress	[168,170]	[165,171,213,214]
heat stress	[140,141]	[19,138,139]
cold tolerance/chilling stress	[86,178,179,182]	[165,166,169]
light stress	[33,34,35,36]	[77,78,135]
shade tolerance	[37,96]	[98]
acid rain impact	[183,184]	[185]
air pollution	[144,173]	[145,146]
soil pollution	[174,175,177]	[147,176]
Biotic Stress		
fungal infection and disease	[187,188]	[195]
viral infection	[174]	[193]
bacterial infection	[189]	[68,194]
insects attack	[190,191]	[192]
mycorrhiza	[198,199]	[196,197]
Foliar Application		
wounding	[201]	
increased crop yields	[202]	
vigor and nutritional status	[203]	[209]
plant growth	[205,206]	[139,204,208]
stress conditions	[207]	[209,212,213,214]
